# Image quality and lesion detectability in simulated [^124^I]MIBG cardiac imaging across 3-generation PET/CT scanners using a phantom model: Implications for activity and acquisition time reduction in clinical practice

**DOI:** 10.1007/s00259-026-08065-0

**Published:** 2026-07-13

**Authors:** Foroud Aghapour Zangeneh, Konstantinos Zeimpekis, Silvano Gnesin, Luisa Maria Knappe, Valentina Garibotto, Christel H. Kamani, John O. Prior, Axel Rominger, Federico Caobelli

**Affiliations:** 1 Department of Nuclear Medicine, Inselspital, Bern University Hospital and University of Bern, Rosenbühlgasse 25/27, 3010 Bern, Switzerland; 2 Institute of Radiation Physics, Lausanne University Hospital and University of Lausanne, Lausanne, Switzerland; 3 Division of Nuclear Medicine and Molecular Imaging, University Hospitals of Geneva, Geneva, Switzerland; 4 Department of Radiology and Medical Informatics, University of Geneva, Geneva, Switzerland; 5 CIBM Center for Biomedical Imaging, Geneva and Lausanne, Switzerland; 6 Department of Cardiology, Lausanne University Hospital, Lausanne, Switzerland; 7 Department of Nuclear Medicine and Molecular Imaging, Lausanne University Hospital, Lausanne, Switzerland

**Keywords:** 124I-MIBG PET/CT, Analog PET, SAFOV PET, LAFOV PET, Cardiac sympathetic imaging

## Abstract

**Background:**

Cardiac sympathetic imaging with iodine-labeled metaiodobenzylguanidine (MIBG) is clinically relevant for assessing autonomic dysfunction and arrhythmic risk in heart failure. The development of positron-emitting ^124^I-MIBG and new-generation PET/CT systems, particularly long axial field-of-view (LAFOV) scanners, may improve image quality and regional defect assessment while enabling protocol optimization. This study compared simulated cardiac ^124^I-MIBG image quality and lesion detectability across three PET/CT generations, analog standard axial field-of-view (SAFOV), digital SAFOV, and LAFOV, and explored the potential for acquisition-time and activity reduction.

**Methods:**

An anthropomorphic torso phantom with cardiac insert was prepared to simulate physiologic ^124^I-MIBG biodistribution. Two configurations were studied: a pathological model with a transmural defect (TD) and a non-transmural defect (NTD), and a normal model with homogeneous myocardial uptake. Acquisitions were performed on three PET/CT systems: analog SAFOV (Biograph mCT), digital SAFOV (Biograph Vision Edge 600), and LAFOV (Biograph Vision Quadra). List-mode data were acquired for 10 min and reconstructed to simulate 1-, 5-, and 10-min acquisitions, yielding nine datasets. Qualitative assessment included lesion detectability and overall image quality scored on 5-point Likert scales by two experienced readers. Quantitative analysis included signal-to-noise ratio (SNR) and contrast-to-noise ratio (CNR). Exploratory pairwise Wald-type comparisons were performed using propagated measurement uncertainty.

**Results:**

LAFOV PET/CT showed the best qualitative and quantitative performance overall. Lesion detectability was highest on LAFOV at all acquisition times, with digital SAFOV intermediate and analog SAFOV lowest. Image quality likewise favored LAFOV, especially at 5 and 10 min. In the pathological phantom, SNR increased from 25.9 to 75.1 on LAFOV, from 18.7 to 41.8 on digital SAFOV, and from 15.5 to 17.1 on analog SAFOV. At 10 min, exploratory uncorrected pairwise comparisons showed nominally higher SNR on LAFOV than on analog SAFOV PET/CT (75.1 vs 17.1, p = 0.028). For the transmural defect, CNR also increased most on LAFOV, with a nominally higher value versus analog SAFOV at 10 min (67.6 vs 14.5, p = 0.044). By contrast, for the low-contrast non-transmural defect, CNR was numerically higher on LAFOV at longer acquisition times, but the absolute inter-scanner differences were smaller and did not reach statistical significance.

**Conclusion:**

LAFOV PET/CT provides superior image quality and lesion detectability for simulated cardiac ^124^I-MIBG imaging and supports shorter acquisitions and potentially lower administered activity. However, the advantage was most pronounced for high-contrast transmural defects, whereas the benefit for low-contrast non-transmural defects was less robust. Reliable detection of subtle partial innervation defects remains challenging and requires dedicated clinical validation.

## Introduction

Iodine-labeled metaiodobenzylguanidine (MIBG) has long been a cornerstone of nuclear medicine for the assessment of neuroendocrine tumors and cardiac sympathetic innervation [1]. Traditionally, [^123^I]MIBG single-photon emission computed tomography (SPECT) imaging has been the clinical standard due to its favorable imaging properties and established diagnostic value [2–4].

Recent advancements in positron emission tomography/computed tomography (PET/CT) technology, particularly the development of the new generation long axial field-of-view (LAFOV) scanners, have introduced significant improvements in imaging capabilities [5–8]. LAFOV PET/CT systems offer enhanced sensitivity, allowing for reductions in radiotracer administered activity and shorter scan durations [6, 9]. Moreover, the extended axial coverage enables whole-body imaging in a single bed position, facilitating comprehensive assessments of disease burden without the need for multiple acquisitions [9–11].

The integration of LAFOV technology with [^124^I]MIBG PET imaging therefore represents a promising approach for improving diagnostic accuracy in both cardiac and oncologic applications (like neuroendocrine tumors) [12] and can be used in treatment planning/dosimetry in patients with thyroid cancer [13]. [^124^I], a positron-emitting isotope with a longer half-life (4.2 days), permits high-resolution imaging and more accurate quantification of tracer uptake. This combination could potentially enhance lesion detectability and characterization, offering a superior alternative to traditional [^123^I] MIBG SPECT imaging.

The primary objective of this study is to evaluate the image quality and lesion detectability using [^124^I] across three PET/CT systems (analog, digital, and LAFOV) using a standardized phantom model. Additionally, we aim to assess the feasibility of reducing in vivo radiation exposure by optimizing imaging protocols and leveraging the enhanced sensitivity of LAFOV PET/CT technology.

## Material and methods

### Phantom setup and acquisition protocols

An anthropomorphic phantom featuring inserts to simulate lungs, liver, left ventricle (LV) wall, LV chamber, and a removable transmural perfusion defect (TD) (Torso Phantom and Cardiac Insert; Data Spectrum Corp.) was used in this study. Activity concentrations of ^124^I were 11 kBq/mL (0.011 MBq/mL) for the LV wall, 17.5 kBq/mL (0.0175 MBq/mL) for the liver, and 0.6 kBq/mL (0.0006 MBq/mL) for the chest compartment and LV chamber, which were used as low-uptake background compartments. The TD insert was filled with non-radioactive water to simulate a transmural defect, whereas the NTD insert was filled with 5.5 kBq/mL (0.0055 MBq/mL), corresponding to half of the LV-wall activity concentration, to simulate a partial innervation defect. Based on these activity concentrations, the LV-wall-to-background ratio, as phantom equivalent of a heart-to-background ratio, was approximately 18.3:1 (11/0.6). Concentrations were selected to approximate the in vivo distribution pattern of iodine-labelled MIBG [3].

Two different configurations were employed: a simulated ^124^I pathological examination (wherein left ventricular wall is indicated as LV1) incorporating both TD and NTD and a normal configuration (wherein left ventricular wall is indicated as LV2), with LV wall uniformly filled with ^124^I.

All regions were segmented using 25% intensity-threshold–based Volumes of Interest (VOI) to ensure identical geometric definitions across acquisitions, using software included in SyngoVia Package (Siemens Healthineers, Knoxville, TN, USA). For each VOI (TD, NTD, LV1, LV2, LV cavity, and background), key PET metrics: mean activity concentration, SD and volume, were extracted.

### PET/CT systems

The tow phantom configurations were acquired on 3 different PET/CT systems: 1) an analog PET/CT scanner with 25 cm standard axial field of view (SAFOV, Biograph mCT, Siemens Healthineers, Knoxville, TN, USA); 2) a digital SAFOV PET/CT system (Biograph Vision Edge 600, Siemens Healthineers, Knoxville, TN, USA) and 3) a LAFOV PET/CT system with 106 cm axial FOV (Biograph Vision Quadra, Siemens Healthineers, Knoxville, TN, USA).

For all the 3 scanners, data were acquired in list-mode over 10 min in a single bed- position. CT-based attenuation correction (AC) using a low-dose CT scan was applied prior to image reconstruction. List-mode data were reconstructed with 1-min, 5-min and 10-min simulated acquisition times across all scanner types, thus generating 9 different datasets: 1-min, 5-min and 10-min acquisition time with analog SAFOV PET/CT system, 1-min, 5-min and 10-min acquisition time with digital SAFOV PET/CT system and 1-min, 5-min and 10-min acquisition time with LAFOV PET/CT system. Alignment between CT and PET images was visually verified. Acquisitions from the 3 different devices have been reconstructed using the same vendor iterative algorithm (Ordered Subset Expectation Maximization) OSEM and TOF (Time-of-Flight) and same reconstruction parameters as summarized in Table 1. Images have been qualitatively and quantitatively evaluated.

**Table 1:** PET image reconstruction parameters

Reconstruction Parameter	Value
Reconstruction algorithm	3D TOF-OSEM
Iterations/Subsets	2/5
Scatter correction	Relative scatter correction
Matrix size	256 × 256 pixels
Post-reconstruction filter	Gaussian, 2 mm FWHM
Attenuation correction	CT-based (CTAC)
Time-of-Flight (TOF)	Yes
Acquisition mode	List-mode
Total acquisition duration	10 min
Simulated acquisition durations	1, 5, and 10 min
Bed positions	1 (single bed position)

Reconstruction parameters were identical across all three PET/CT. Simulated acquisition durations of 1, 5, and 10 min were derived from the 10-min list-mode acquisition by temporal sub-sampling

Abbreviations: *SAFOV* Standard Axial Field of View; *LAFOV* Long Axial Field of View; *TOF* Time-of-Flight; *OSEM* Ordered Subset Expectation Maximization; *3D* three-dimensional; *CTAC* CT-based Attenuation Correction; *FWHM* Full Width at Half Maximum

### Qualitative assessment

Image quality was assessed visually using two subjective metrics: 1) Lesion detectability, reflecting the visual conspicuity of perfusion defects and 2) overall image quality (IQ), reflecting global image interpretability. Both metrics were scored by two experienced nuclear medicine physicians blind to dataset configuration on a 5-point Likert scale for each of the 9 datasets. Discrepancies were resolved by consensus.

### Quantification metrics

Quantitative analyses were based on mean activity concentrations (kBq/mL) extracted from predefined volumes of interest (VOIs). All VOIs were defined semi-automatically using syngo.via (Siemens Healthineers, Knoxville, TN, USA). Structural VOIs were defined for the pathological myocardium (LV1), the normal homogeneous myocardium (LV2), the transmural defect (TD), and the non-transmural defect (NTD). These VOIs were placed at the known anatomical locations of the corresponding phantom compartments and delineated using a fixed 25% iso-contour threshold relative to the maximum voxel value within the respective region. For the myocardial VOIs, placement was centered in the mid-ventricular LV wall, whereas the TD and NTD VOIs were centered within the corresponding defect regions. From each VOI, mean activity concentration, standard deviation (SD), and volume were extracted.

To minimize operator- and reconstruction-dependent variability, each VOI was defined once and then copied identically across all nine reconstructed datasets. Thus, VOI location, size, and geometry were held constant across scanners and acquisition times, ensuring that inter-dataset differences reflected scanner- and acquisition-dependent effects rather than differences in VOI placement or segmentation.

#### Signal-to-noise ratio (SNR)

SNR was calculated separately for the pathological and normal myocardial configurations as follows:

$${SNR}_{LV1}= \frac{{\mu}_{LV1}}{{\sigma}_{BG}}, \hspace{1em}{SNR}_{LV2}= \frac{{\mu}_{LV2}}{{\sigma}_{BG}}$$

where ${\mu}_{LV1}$ and ${\mu}_{LV2}$ denote the mean activity concentration in pathological and normal myocardium, respectively, and ${\sigma}_{BG}$ the SD of activity concentration within a background (BG) VOI.

#### Contrast-to-noise ratio (CNR)

Lesion contrast was quantified using CNR for both defect types as follows:

$${CNR}_{LV1-TD}= \frac{{\mu}_{LV1}- {\mu}_{TD}}{{\sigma}_{BG}},\hspace{1em}{CNR}_{LV1-NTD}= \frac{{\mu}_{LV1}- {\mu}_{NTD}}{{\sigma}_{BG}}$$

where μTD and μNTD denote the mean activity concentration within the transmural and non-transmural defect VOIs, respectively. These metrics reflect the detectability of defects of varying severity relative to the pathological myocardial reference VOI.

#### Background definition and noise estimation

The background VOI was defined separately as a fixed 1 cm^3^ spherical VOI placed within the homogeneous low-uptake left-ventricular cavity. No iso-contour thresholding was applied to the background VOI, because threshold-based segmentation in a low-uptake region would bias the noise estimate. The SD of the activity concentration within this background VOI (σBG) was used as the noise term for all SNR and CNR calculations.

### Statistical analysis

Objective phantom endpoints were analyzed with prespecified exploratory pairwise Wald-type comparisons based on propagated measurement uncertainty. For each derived metric, uncertainty was propagated from the underlying ROI-level SDs by first-order error propagation. The planned contrasts addressed 1) acquisition-time differences within each scanner, 1 vs 5 min, 1 vs 10 min, and 5 vs 10 min, and 2) scanner differences within each acquisition time, analog SAFOV PET/CT vs digital SAFOV PET/CT, analog SAFOV PET/CT vs LAFOV PET/CT, and digital SAFOV PET/CT vs LAFOV PET/CT. All pairwise comparisons were prespecified as exploratory and hypothesis-generating. No correction for multiple comparisons, such as Bonferroni, Holm, or false-discovery-rate correction, was applied. This decision reflects the technical phantom-study design, in which endpoint estimates were derived from a single phantom experiment with propagated measurement uncertainty rather than from independent biological replicates. The purpose of the pairwise comparisons was therefore to support the descriptive interpretation of scanner- and acquisition-time trends rather than to provide confirmatory statistical inference. Accordingly, all p-values are reported descriptively, and nominal p-values below 0.05 should be interpreted cautiously. For each contrast, the test statistic was calculated as z = (× 2 − × 1)/sqrt(u1^2^ + u2^2^), where *x* denotes the endpoint estimate and *u* its propagated uncertainty, with two-sided p-values obtained from the standard normal distribution. Objective data are shown as estimate ± propagated uncertainty. Subjective ordinal scores were summarized descriptively.

## Results

### Qualitative assessment

**Fig. 1:**
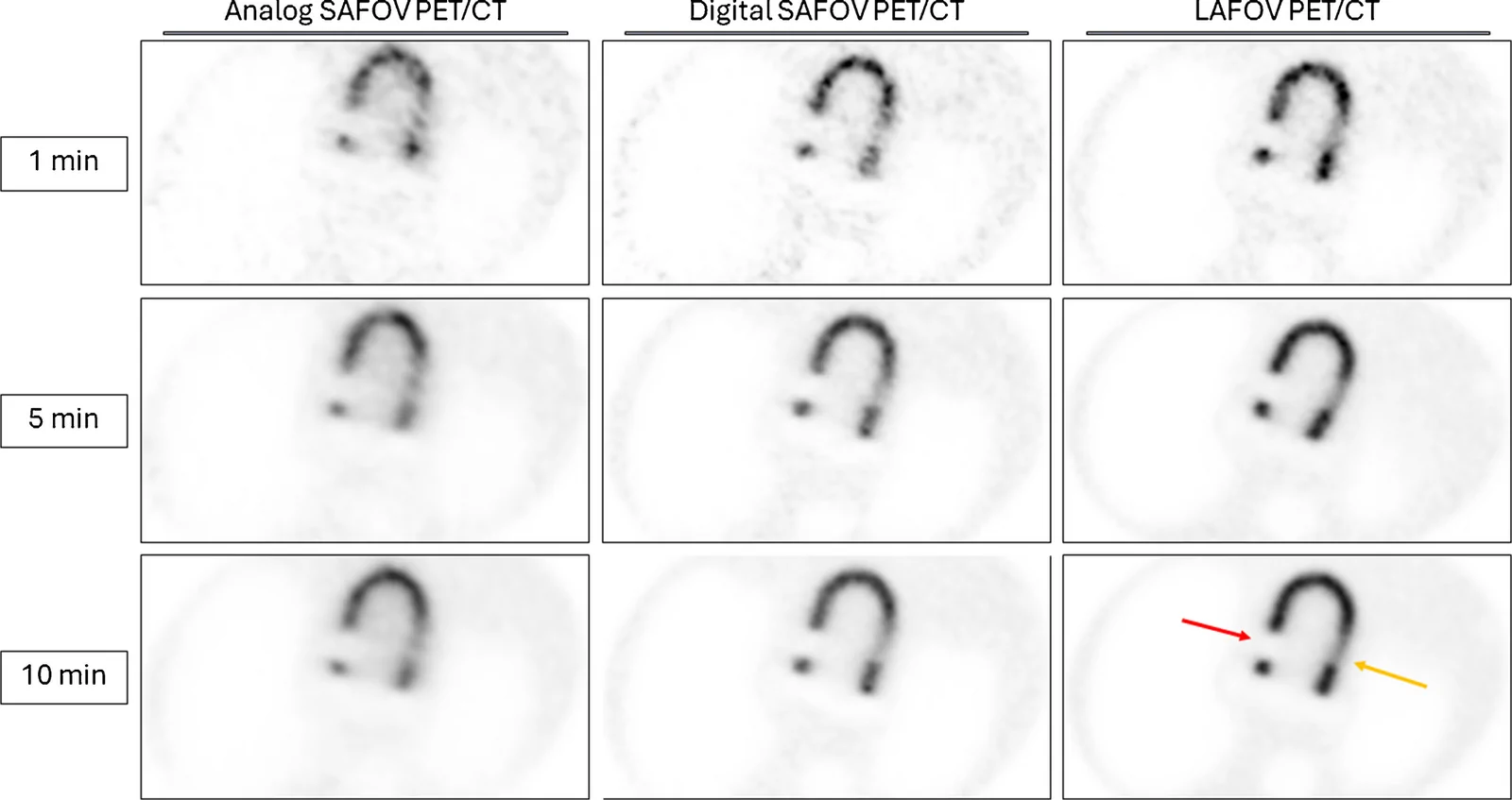
Representative images highlighting the different image quality across different PET/CT scanners and acquisition time, in the simulated pathological phantom study, featuring a transmural defect (TD, red arrow) and a non-transmural defect (NTD, yellow arrow)

Subjective image quality metrics showed consistent visual differences across scanners and acquisition times (Fig. 1). Visual inspection of the reconstructed images demonstrated a clear tendency toward higher image quality and better lesion conspicuity with digital PET/CT systems and with longer acquisition times, with the most favorable visual appearance on LAFOV PET/CT, particularly at acquisition times of at least 5 min.

**Fig. 2:**
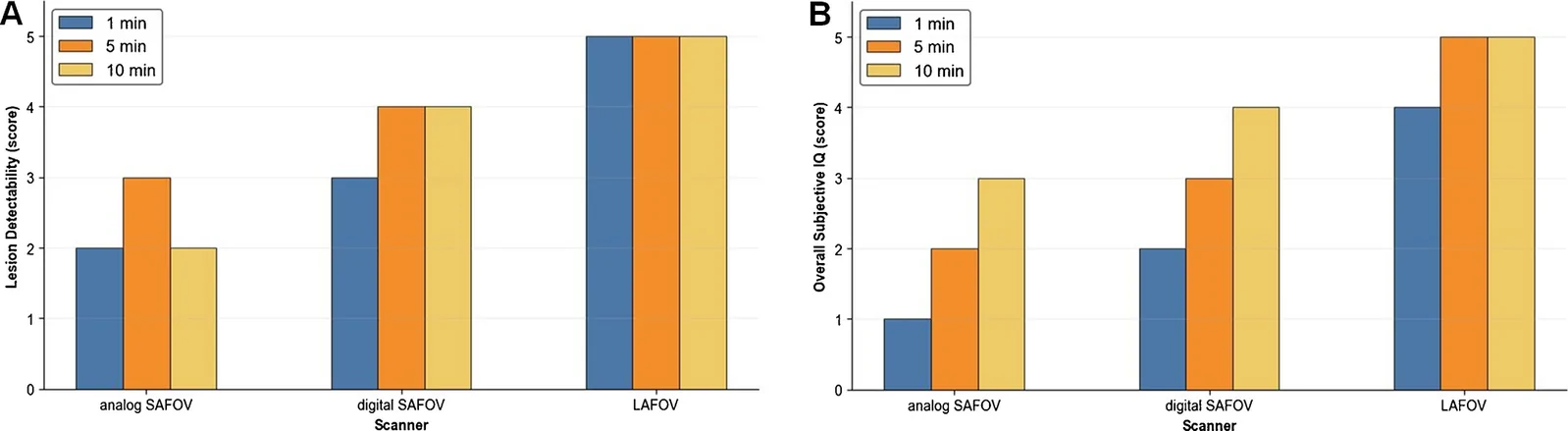
Detectability score (**A**) and overall image quality score (**B**) across different PET/CT scanners and acquisition times

Lesion detectability scores (Fig. 2A) were highest for LAFOV across all acquisition durations (score 5), indicating consistently excellent visual conspicuity of perfusion defects. Digital SAFOV showed intermediate detectability (scores 3–4), which improved with increasing acquisition time, whereas detectability scores were lowest (2–3) for analog SAFOV PET/CT, particularly at shorter acquisition times.

Overall image quality followed a similar pattern (Fig. 2B). LAFOV achieved the highest scores (4–5), with only minor variation across acquisition times. Digital SAFOV PET/CT showed intermediate image quality (scores 2–4), which improved with longer acquisition time, while analog SAFOV PET/CT yielded the lowest scores (1–3). Overall, the qualitative assessment consistently favored LAFOV PET/CT, with digital SAFOV PET/CT ranking intermediate and analog SAFOV PET/CT lowest.

### Quantitative assessment

#### Signal-to-noise ratio

**Fig. 3:**
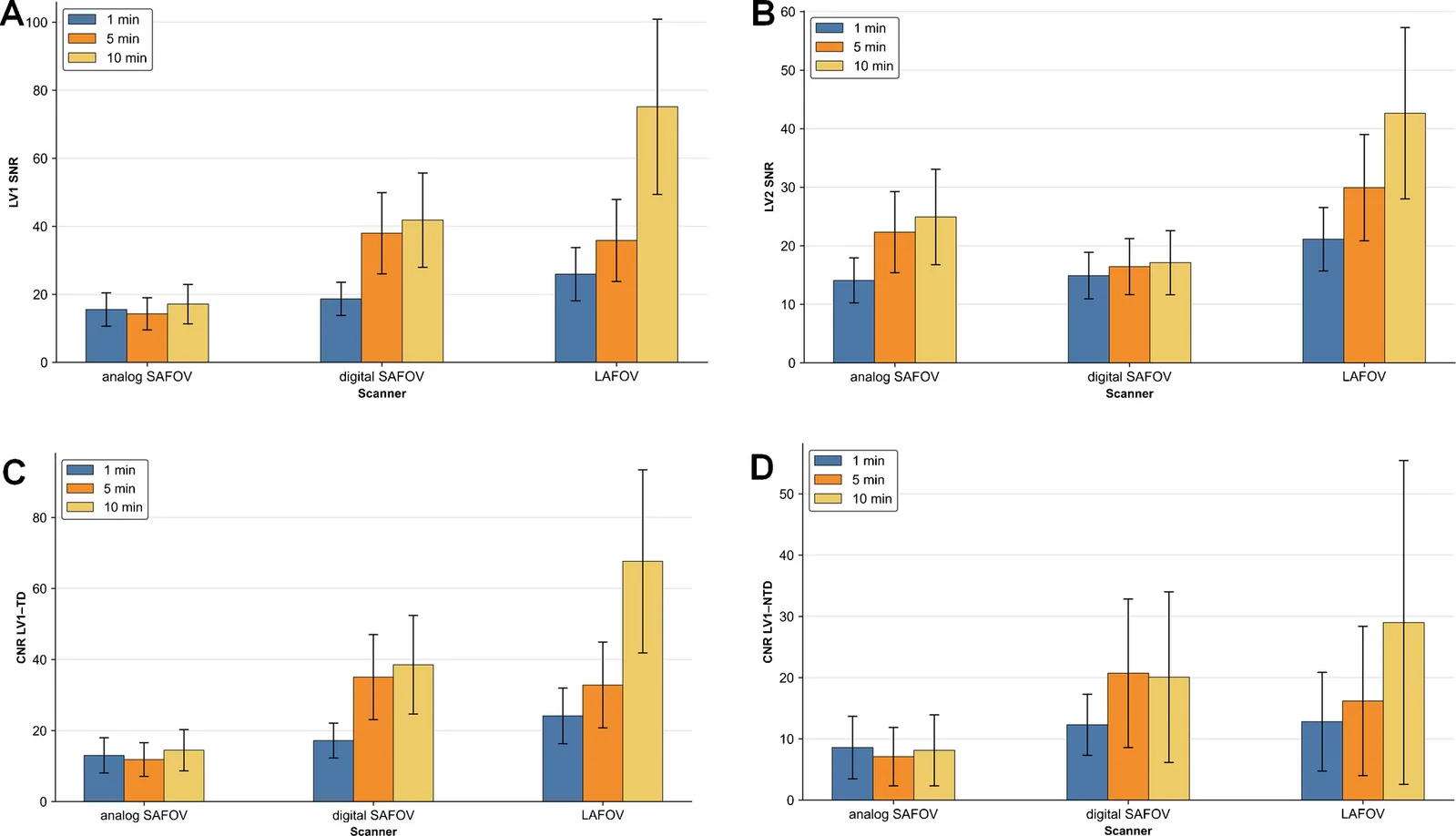
Quantitative evaluation across different PET/CT scanners and acquisition times. **A**: signal-to-noise ratio (SNR) calculated in the simulated pathological phantom study (LV1). **B**: signal-to-noise ratio (SNR) calculated in the simulated normal phantom study (LV2). **C**: contrast-to-noise ratio (CNR) for the transmural defect (TD). **D**: contrast-to-noise ratio (CNR) for the non-transmural defect (NTD). Error bars represent propagated measurement uncertainty derived by first-order error propagation from the VOI-level standard deviations. SAFOV = standard axial field of view; LAFOV = long axial field of view

SNR increased with longer acquisition times for all 3 scanners. In the simulated pathological cardiac [^124^I]MIBG study, LAFOV PET/CT demonstrated the most marked increase, rising from 25.9 at 1 min to 75.1 at 10 min (Fig. 3A). The digital SAFOV PET/CT showed a moderate increase (18.7 to 41.8), whereas analog SAFOV PET/CT exhibited comparatively lower SNR values with only limited gain over time (15.5 to 17.1). At 10 min, exploratory uncorrected pairwise comparisons showed nominally higher SNR for LAFOV than for analog SAFOV PET/CT (75.1 vs 17.1, p = 0.028).

A similar trend was observed in the simulated normal [^124^I]MIBG study. LAFOV consistently provided the highest SNR across all acquisition times (21.1, 29.9, and 42.6 for 1, 5, and 10 min, respectively), followed by analog SAFOV PET/CT and digital SAFOV PET/CT (Fig. 3B). Digital SAFOV PET/CT showed only modest improvement with increasing acquisition time, whereas analog SAFOV PET/CT demonstrated a more marked increase between 1 and 10 min.

#### Contrast-to-noise ratio

Considering the TD, CNR values improved substantially with increasing acquisition duration for all scanners. CNR increased markedly on all systems (Fig. 3C). LAFOV PET/CT exhibited the highest contrast at longer durations, rising from 24.1 at 1 min to 67.6 at 10 min. Digital SAFOV PET/CT showed intermediate values (17.2 to 38.5), while analog SAFOV PET/CT demonstrated the lowest CNR overall (13.0 to 14.5), with only limited improvement beyond 5 min. At 10 min, exploratory uncorrected pairwise comparisons showed nominally higher CNR for LAFOV than for analog SAFOV PET/CT for the TD (67.6 vs 14.5, p = 0.044).

For NTD, CNR also increased with longer acquisition time, but with less pronounced absolute differences (Fig. 3D). LAFOV PET/CT, again, demonstrated the highest values at longer acquisition times (12.8 to 29.0), followed by digital SAFOV PET/CT (12.3 to 20.1), whereas analog SAFOV PET/CT remained consistently lower (8.6 to 8.1). Compared with TD, CNR values for NTD were reduced across all systems, reflecting the lower intrinsic contrast of these lesions. No statistically significant pairwise differences were observed for CNR. These findings indicate that the LAFOV advantage for the non-transmural defect should be interpreted primarily as a descriptive numerical trend. In contrast to the TD, the lower intrinsic contrast of the NTD limited both the absolute CNR gain and the magnitude of inter-scanner differences.

#### Background noise

Background noise analysis revealed clear differences between PET systems and acquisition durations. For 1-min acquisitions, the digital SAFOV system exhibited the highest background variability, whereas the analog SAFOV PET/CT system showed the lowest SD, indicating reduced statistical fluctuation in low-uptake regions despite its lower sensitivity. The LAFOV system demonstrated intermediate background noise levels. These findings are consistent with the interplay between detector sensitivity, prompt-gamma contamination, and reconstruction stability in short [^124^I]MIBG acquisitions.

## Discussion

Autonomic dysfunction is tightly linked to heart failure progression and arrhythmogenesis, which makes cardiac sympathetic imaging clinically relevant. Large clinical studies with [^123^I]MIBG have shown that neuronal imaging adds prognostic information beyond conventional functional parameters, showing a strong association with regional innervation defect burden and cardiac events [14–16]. Furthermore, the extent of PET-defined sympathetic denervation in the PAREPET study [17] predicted sudden cardiac arrest independently of LV ejection fraction (EF) and infarct volume, with the highest denervation tertile showing the greatest arrhythmic risk. Accordingly, a regional and quantitative approach to innervation imaging is likely to be more informative than a purely global semiquantitative readout when the clinical question concerns arrhythmic substrate rather than heart failure severity alone [18, 19].

In this context, PET is more attractive than SPECT, and more recently, ^18^F-labelled tracers such as flubrobenguane and fluorohydroxyphenethylguanidines have been developed, which combine favorable kinetics with the possibility of a broader dissemination of quantitative PET innervation imaging [18–20]. Within this evolving landscape, [^124^I]MIBG represents an interesting option because it preserves the MIBG molecular scaffold while enabling PET-based assessment, and early clinical experience has already shown its feasibility for high-resolution imaging in the oncological setting [12]. However, the established diagnostic and prognostic evidence for cardiac MIBG imaging derives primarily from [^123^I]MIBG SPECT, and cannot be assumed to translate directly to [^124^I]MIBG PET without dedicated cardiac patient studies. At the same time, radiation exposure cannot be ignored. The most recent dosimetry analysis reported an adult effective dose coefficient of 0.25 ± 0.04 mSv/MBq for [^124^I]MIBG, clearly higher than the projected coefficient for [^123^I]MIBG (0.014 mSv/MBq) and therefore a relevant consideration when cardiac protocols are translated into clinical routine [21].

New-generation PET systems are expected to improve cardiac innervation imaging because both digital detector technology and extended axial coverage increase sensitivity and effective count collection. Head-to-head clinical LAFOV experience has shown that image quality and lesion quantification comparable to digital SAFOV PET can be achieved in markedly shorter scan times, while recent reviews have highlighted how this sensitivity gain can be exchanged for either faster acquisition or lower administered activity [5, 10]. This technical background is highly relevant for [^124^I]MIBG, where the need for regional, quantitative imaging must be balanced against radiation exposure.

To our knowledge, however, no previous study has systematically compared analog SAFOV, digital SAFOV, and LAFOV PET/CT specifically for ^124^I and [^124^I]MIBG cardiac sympathetic imaging. Most available data concern pairwise comparison in the oncological setting or concerns general LAFOV performance, whereas the current literature on cardiac innervation imaging has focused on planar or SPECT approaches or PET tracers other than [^124^I]MIBG [5, 10, 18–20]. In this context, our work provides novelty in that it offers a side-by-side technical comparison of 3 PET generations for simulated cardiac [^124^I]MIBG imaging, integrating quantitative metrics, exploratory pairwise statistics, and blinded visual assessment within the same phantom framework.

Our findings are in line with previous LAFOV PET studies using other radiotracers, which have consistently shown that the increased system sensitivity of extended axial field-of-view scanners can be translated into shorter acquisition times and/or reduced administered activity while preserving image quality [5, 9, 10]. In cardiac imaging, early experience with dynamic whole-body ^82^Rb PET on LAFOV PET/CT demonstrated the feasibility of acquiring myocardial perfusion information with extended axial coverage [8], and more recent clinical data have suggested improved accuracy of functional cardiac PET parameters at low administered ^82^Rb activity on LAFOV systems [22]. Similarly, dynamic ^18^F-FDG applications and head-to-head comparisons with digital SAFOV PET/CT have shown that comparable image quality and quantitative information can be obtained at substantially shorter scan durations [5, 10, 23].

The present study extends this principle to simulated cardiac [^124^I]MIBG imaging. This is relevant because [^124^I] differs from conventional positron emitters such as ^1^⁸F and ⁸^2^Rb due to its prompt-gamma emissions, which may introduce cascade coincidences and affect quantitative accuracy. Accordingly, our results suggest that the LAFOV sensitivity advantage is preserved mainly in noise-normalized metrics, such as SNR and CNR, and in visual image quality, whereas mean-value-based ratios may show more complex behavior. Thus, our data are consistent with previous LAFOV experience while also emphasizing the need for radionuclide-specific optimization when translating acquisition protocols across PET technologies.

In the present study, LAFOV PET/CT consistently provided the best overall performance for subjective image quality, lesion detectability, SNR, and CNR. Digital SAFOV PET/CT ranked intermediate, whereas analog SAFOV PET/CT was the most noise-limited system. This hierarchy was mirrored by the qualitative evaluation, in which LAFOV PET/CT preserved sharper myocardial contours and clearer delineation of defects already at 1 min and most consistently at 5–10 min. Digital SAFOV PET/CT improved with longer acquisition time, whereas analog SAFOV PET/CT remained visually more blurred. However, the magnitude of this advantage depended on lesion contrast: it was most robust for the transmural defect, whereas the non-transmural defect remained more challenging because of its intrinsically lower contrast. The NTD deserves separate consideration because it represents a lower-contrast and therefore more clinically demanding target. In our phantom, the NTD retained half of the myocardial activity, whereas the transmural defect contained no activity. Consequently, the lesion-to-myocardium contrast and the achievable CNR were intrinsically lower for the NTD than for the TD. Although LAFOV PET/CT numerically improved NTD CNR at longer acquisition times and visually improved defect conspicuity, inter-scanner differences for NTD-CNR did not reach statistical significance. Therefore, the benefit of LAFOV PET/CT appears most robust for higher-contrast defects, while reliable detection of subtle partial innervation defects remains challenging and should be specifically evaluated in clinical studies.

These findings support not only shorter acquisitions, but also a realistic strategy of activity de-escalation. The phantom concentrations used here were selected to reproduce a physiologic cardiac MIBG biodistribution. On that basis, considering that standard adult [^124^I]MIBG activities are currently around 40 MBq [21], our data suggest that a dose reduction by at least 50% may be a reasonable target if acquisition time is prolonged, particularly on LAFOV PET/CT systems, where the gain with longer acquisitions was most pronounced in our phantom study. To provide an approximate estimate of potential activity reduction, we applied an SNR-based scaling approach, assuming that SNR scales with the square root of administered activity. For the pathological myocardial VOI, SNR was 25.9 for 1-min LAFOV, 17.1 for 10-min analog SAFOV, and 41.8 for 10-min digital SAFOV. Using a reference activity of 40 MBq, a 1-min LAFOV acquisition would require approximately 17.4 MBq to match the SNR of a 10-min analog SAFOV acquisition, corresponding to a theoretical activity reduction of approximately 56% and an effective dose of approximately 4.4 mSv, based on an effective dose coefficient of 0.25 mSv/MBq. Conversely, matching the 10-min digital SAFOV SNR would require approximately 104.2 MBq, indicating that activity reduction relative to digital SAFOV is not supported for this specific 1-min LAFOV SNR endpoint. These estimates are phantom-based and should be interpreted as approximate image-quality calculations rather than clinical dose recommendations. This view is also consistent with prior phantom work showing that [^124^I]MIBG PET/CT may improve image quality and/or permit dose-efficient protocols also compared with [^123^I]MIBG SPECT/CT [24].

The more complex behavior of mean-based ratios in our study also has a plausible physical explanation. ^124^I is not a pure positron emitter, and prompt gamma emissions can generate cascade coincidences and additional background events that degrade quantitative accuracy. As discussed recently in the work by Pratt et al. [25] the impact of these prompt-gamma-related events depends on how they are handled by the acquisition and reconstruction chain. In our setting, the higher sensitivity of LAFOV PET/CT is beneficial for true-count statistics and therefore for SNR, CNR, and visual detectability, but it may also increase the registration of prompt-gamma-related events. This likely explains why LAFOV PET/CT was clearly superior in noise-normalized and qualitative metrics, whereas some mean-value ratios showed a more nuanced pattern.

Overall, the present findings support a technical rationale for further exploring [^124^I]MIBG PET, particularly when combined with LAFOV technology, as a platform for regional and quantitative cardiac sympathetic imaging. The potential clinical utility of this approach lies in the combination of PET-based regional assessment, improved count sensitivity, and the possibility of shorter and/or lower-activity protocols. These features may be relevant in future patient-centered applications in which regional denervation, rather than only global tracer retention, is expected to contribute to risk stratification, especially for arrhythmic events. However, the present phantom study evaluates image quality and lesion detectability only. It does not establish diagnostic or prognostic superiority of [^124^I]MIBG PET over established [^123^I]MIBG SPECT, for which clinical evidence already exists. The incremental diagnostic and prognostic value of [^124^I]MIBG LAFOV PET therefore remains to be demonstrated in dedicated clinical studies with patient outcomes.

### Limitations

Some limitations to the present study should be acknowledged. First, we did not evaluate the influence of other iterative reconstruction parameters (i.e., number of subsets, iterations, and resolution recovery) on the results. Second, these results were obtained in an ideal standard situation and might not be completely applicable in clinical practice; therefore, further clinical studies on patients are necessary to validate the clinical relevance of the findings. Furthermore, only one phantom geometry and one activity distribution were evaluated. This fixed configuration was deliberately chosen to keep geometry, activity concentration, and defect contrast constant across scanners and acquisition times, thereby enabling an unbiased head-to-head comparison of the three PET/CT systems. Nevertheless, different body sizes, activity concentrations, defect contrasts, and defect geometries may influence image quality, SNR, CNR, and lesion detectability. Future studies should therefore evaluate whether these findings generalize to additional phantom configurations and, more importantly, to clinical patient data. In addition, although VOI location and size were controlled by design through identical VOI propagation across all reconstructions, we did not perform a dedicated VOI-perturbation or inter-reader variability analysis; therefore, the impact of alternative VOI placements or sizes should be assessed in future clinical studies. Finally, the phantom used in this study only allows the evaluation of ungated PET images, and the impact of motion cannot be evaluated. Nevertheless, phantom-based analyses provide a controlled framework for system comparison and protocol optimization and remain essential for understanding scanner-dependent performance characteristics.

## Conclusion

LAFOV PET/CT provided the best overall performance for simulated ^124^I-MIBG cardiac imaging, with higher qualitative scores and favorable SNR and CNR compared with conventional systems. Notably, 1-min LAFOV acquisitions achieved image quality comparable to, and in some settings better than, longer acquisitions on conventional scanners, supporting the feasibility of marked reductions in acquisition time and potentially administered activity. However, the advantage was most pronounced for high-contrast transmural defects. For the low-contrast non-transmural defect, CNR was numerically higher on LAFOV at longer acquisition times, but inter-scanner differences were smaller and did not reach statistical significance. Thus, subtle partial innervation defects remain challenging. These findings are based on a phantom model and should therefore be considered preclinical. Prospective clinical validation in patients is required before protocol or activity reductions can be translated into routine clinical practice.

## Data Availability

The datasets generated during the current study are available from the corresponding author on reasonable request.
